# Exploring the Experiences of Living With Stroke Through Narrative: Stroke Survivors’ Perspectives

**DOI:** 10.1177/2333393616646518

**Published:** 2016-05-05

**Authors:** Nasrin Nasr, Susan Mawson, Peter Wright, Jack Parker, Gail Mountain

**Affiliations:** 1 University of Sheffield, Sheffield, United Kingdom; 2 National Institute for Health Research, Sheffield, United Kingdom; 3 Newcastle University, Newcastle upon Tyne, United Kingdom; 4 Bradford University, Bradford, United Kingdom

**Keywords:** embodiment/bodily experiences, care, long term, illness and disease, experiences, interviews, lived experience, narrative inquiry, relationships, research, qualitative, stroke

## Abstract

Chronic illness models are normally used to explain and predict the experience of living with a long-term condition. The aim of this study was to present the findings of narrative interviews with stroke survivors and their family carers to understand their experiences of stroke. We interviewed five people with stroke and three family carers from the United Kingdom. We used thematic analysis to generate themes from their narrative accounts and then linked them to broader theoretical perspectives while influenced by the concept of reinterpretation of life. The narrative accounts of participants are mainly structured based on how their changed bodies poststroke changed their identities and roles and consequently their relationships with others. In this study, we underline the need for using methods like narrative to explain strategies that people use to make sense of their experiences of living with a long-term condition such as stroke.

## Background

Chronic illness models are normally used to explain and predict the experience of living with a long-term condition. Exemplified by the Chronic Disease Self-Management Programs (Lorig & Holman, 2003), these models are also used worldwide as a framework to design self-management programs to respond to the challenges such as an aging population and increasing chronic diseases. Current approaches to self-management are often based on normative descriptions of the standards, values, and understandings of the illness experience among chronically ill patients, while training and education remain at the heart of such programs and the expert patient is defined implicitly as a “responsible individual” (Kendall, Ehrlich, Sunderland, Muenchberger, & Rushton, 2011) who is able to make appropriate use of resources. These approaches fail to address the more complex aspects of living with a chronic illness as depicted mostly by qualitative research, for example, patients’ experiences of bodies and how the changed bodies affect the construction of new identities, roles, and their relationships with self and others following a chronic illness (Kvigne & Kirkevold, 2003).

Corbin and Strauss’s (1992) model is used to develop self-management programs and is based on assumptions that there are some commonalities in the experience of chronic illness shared by all patients during the course of the disease. In other words, the model provides a prospective vision of the disease trajectory based on professionals’ knowledge and patients’ experiences, standards, and values (Burton, 2000). It involves three tasks that people with long-term conditions normally deal with including caring for the disease such as medical management of their condition, maintaining, revising, or changing life goals such as doing chores and maintaining hobbies, and dealing with negative emotional feelings such as anger and depression (Lorig, 1993).

Another chronic illness model is Becker’s (1993) continuity theory. It establishes a link between creating a sense of continuity and routine daily activities, which requires additional attention to the restoration of old daily activities or replacing them with the new identified ones. In this theory, the meaning of continuity is partly linked to the objective factors of function and to the continuation or restoration of functional abilities, though a sense of continuity may be fulfilled by shifting the values to other aspects of life that helps maintain a sense of identity regardless of change.

Biographical disruption (Bury, 1982) as the most influential interpretive approach for long-term conditions provides another alternative model to explain the trajectory of a long-term condition. According to the model, the experience of a chronic illness is formed both by the physical burdens of the disease within the social context and by how the individual and others who perceive those burdens. Bringing others to the picture, Bury explains a testing situation in which the individual compares the meanings attached to their stressful situation to what they see as the reality of their life and calls it a situation of “meanings at risk” because what counts as a meaningful situation for them may not be shared by others. Following the onset of the disease, the individual attempts to explain the long-term consequences of the disease with the help of medical knowledge to repair disruptive life stories. Patients’ expectation of these interventions and their effectiveness, however, may change over the course of the disease (Bury, 1991).

The purpose of this study was to use a pragmatic approach to explore how stroke survivors as “expert” in their own experiences perceive their experiences of living with stroke and how context-dependent components of human experience can be understood.

The aim of this article is to present the qualitative findings from narrative interviews with stroke survivors and their carers to examine their experiences of stroke and their thoughts toward managing their condition. The data collected from participants depicted the complexity of stroke that goes to the heart of their life world with potential to support the design of a self-management program.

## Method

### Study Design

We employed a qualitative research design and used narrative interviews (Mishler, 2006; Riessman, 2002) to allow participants to express their experiences of living with stroke freely. In this approach, meanings are constructed contextually and interactionally (Mishler, 1986). Narrative inquiry supports the dynamic processes of storytelling through which an account is generated as a result of an interaction between an interviewer and a participant within a specific context. It is evident that knowledge in personal life stories enriches our understanding of the impact of illness and disease on people’s lives (Frank, 1995). Through narrative, people revise their experiences of illness, change their values, and reinterpret the meaning of life based on their new perceived situations (Mishler, 2006). As a result, meaningful narrative and dialogue as central properties of value-based knowledge (Flyvbjerg, 2001) can explain strategies that people adopt to face the demands of living with a chronic illness.

Of particular relevance here is Bakhtin’s “dialogical” approach “dialogism” (Baxter, 2004; Holquist, 1990). “Dialogism” uses language as a model or metaphor to understand human relationships and experience. The focus is on how we make sense of our world, ourselves, and others, and how we act in the world as concerned individuals. A dialogical approach to knowing takes as its starting point the idea that to “know” what another person is experiencing or thinking requires a particular form of empathic act on our part. We can never fully understand someone’s experience by adopting the stance of an impartial, neutral observer. An empathic approach requires us to try to understand the other as a unique individual, or center of value, with a past and a future in process. Furthermore, because we can never truly act toward another as an impartial observer, we are always forming a response to that other from a value position which is our own. This process thus requires us to enter into a relationship with the other by giving something of ourselves. It does not mean that we simply fuse with “the other,” rather it requires us to be able to see the world as the other sees it while maintaining one’s own identity and center of value. Bakhtin called this creative understanding, because it is in recognizing the gap between these different perspectives that new possibilities and new meanings emerge from which both sides can learn. For Bakhtin, such understanding was only possible when both sides saw themselves as equal but differently placed centers of value. Each expert in their own experiences but capable of seeing and learning from the other. In this study, meanings were constructed as a result of interaction and dialogue between stroke survivors as expert patients and researchers. Co-construction of meaning between the researcher and the participants happened through the method of narrative interviewing. The purpose of life story interviews is to provide the participants with the option to tell their stories in their preferred ways. We did not have a list of fixed questions prior to the interview. The story was constructed over the course of the interview interactionally. Through reframing the questions and answers continually, the researcher and the participant reached the point where they had the same understanding of meanings. The narrative questions were part of an iterative process through which their meaning and that of their answers were created in the interaction (Mishler, 1986, 1997).

Individual patients are physically immersed in their experiences and their skills become embodied (Dreyfus & Dreyfus, 1986; Williams & Popay, 2001). The gap between professional expertise and lay expertise is, however, better perceived and integrated when dialogue is established. In other words, it is possible to become fluent in the language of “expert patients” without ever experiencing a disease itself physically by being engaged in interactional expertise (Collins & Evans, 2007), as it is explained in narrative inquiry.

We applied Riessman’s (1993) five levels of narrative inquiry to design the study. The first level is “attending to experience.” It is the immediate experience as an individual lives it and makes meaning out of that event. Then, at Level 2, the narrator transfers the actual experience and its meaning into words by talking and listening. The third level is transcribing and translating the spoken language into a text, just like a photographer who uses the photography technology to create varied versions of the same object. The process of interpretation that has already started in the transcription level continues in the fourth level to analyze the experience. Through the process of analysis, the researcher sums up the identified similarities in narrative accounts and recreates an abstract that is different from the oral stories of individuals (Riessman, 1993). Reading experience is the final stage in Riessman’s levels of representation.

### Participants

Following ethical approval from the local National Health Service (NHS) research ethics committee (Reference No. 08/H1306/46), participants were recruited from local stroke clubs, community and day rehabilitation centers, and outpatient rehabilitation units. To facilitate recruitment of people with stroke and their carers, initial meetings were held with managers of identified stroke centers. At these meetings, the study was explained to the staff and they were provided with information about the study inclusion criteria. Information and forms for potential participants to express interest in participation and to give consent for their contact details to be passed on to the research team was provided. The researchers’ contact information was given to the staff to be passed on to those who come forward. Potential participants were approached by the staff and those who agreed to be contacted were visited by researchers at a venue of their choice where the study was explained to them. The inclusion criteria were that those recruited should have a diagnosis of stroke, be capable of giving informed consent, have adequate English language skills to understand and express themselves verbally, and be living in the community. Five persons with stroke and three carers provided written informed consent before they were interviewed.

### Data Collection

Nasrin Nasr conducted narrative interviews with the stroke survivors in their own homes or if they preferred the interview also involved their carers. We used narrative inquiry to facilitate a holistic approach to understanding participants’ experiences of stroke as a whole. The interviews started with an open question that prompted the participant to talk freely about their experiences of stroke. The question aimed at inducing narrative: “Please tell me about your experience of stroke, start wherever you like and take the time you need” (Wengraf, 2001, p. 127). Participants were not interrupted and the interviewer only sought clarification on the themes that were raised in their accounts. Interviews were tape recorded with the participants’ permission and then transcribed verbatim.

### Data Analysis

We used Coffey and Atkinson’s (1996) model of thematic analysis to summarize the interview accounts, section the data, and code the segmented data. The model includes two main phases: de-contextualization and re-contextualization. First, themes were identified in individual narrative accounts and were related to the interview language of the participant (de-contextualization). Then, the narrative accounts were compared and similarities and differences were examined. As a result, categories were generated that were related to the research or theoretical language of the study. The theoretical categories introduced new contexts and moved the accounts to the wider analytical prospects (re-contextualization). The analysis was influenced by the concept of reinterpretation of life and the metaphor “double arrow of time” where “the past is not set in stone, but the meaning of events and experiences is constantly being reframed within the contexts of our current and ongoing lives” (Mishler, 2006, p. 30).

## Results

Five people with stroke and three family carers took part in the study. From the participants’ narrative accounts, we can compile four main themes.

### Normalizing the Condition

The feature of the stroke was very important to the participants as they provided narrative account on the cause and nature of the stroke. Their shared perception was that the stroke hit them suddenly. A 65-year-old retired English teacher recalled how it felt when she had the stroke:

I suddenly felt funny and I knew it was serious, the weirdest thing. I knew it was very serious and I remember thinking it’s not a heart attack because I’ve got no pain in me chest. . . . and they knew what it was as soon as I got to the hospital because I was a smoker and I was quite young and quite fit. (Stroke survivor)

The following statement was provided by a carer:

Yes, stroke always happens very suddenly. In this case totally out of blue and he (her husband) was as far as we could say in perfect health, no problems and you know when stroke came it was you know very severe. (Carer)

Stroke was perceived by the participants as a sudden disruptive event; however, they needed to normalize it to fit into the context of their social life. Some participants put the stroke within the process of aging and used the factor of age to normalize the stroke condition. A 74-year-old retired shop manager unfolds her experience of stroke embedded in her life events and the limitations caused by old age as a normal process:

I would like doing things for other people but I can’t do anything you know. Life is not as good as when you’re forty obviously. (Stroke survivor)

However, for some of the participants, the stroke was not associated with old age and their attempt was to avoid the situations in which they would be identified as old. For example, a stroke survivor described her thoughts of joining a lunch club:

I joined a couple of lunch clubs but they are very old people and sometimes I think should I be here? Although I’m out of the house and I get good meal and I’m talking to people, I think I’m not sure this is me, I don’t want to be all the time with old people because I’m not old, I’m not old in here (pointing to her head). (Stroke survivor)

In the following quotation, she describes her poststroke body in a more familiar and usual context:

I’d like to get slimmer. I’ve put weight on because I’m not active enough. I’ve always been slim because I’ve always been running about active so my self-image is poor because I see fat. I’m fatter two stones (28 pounds), heavier than I was before well but then I smoked. (Stroke survivor)

Another carer tries to understand her partner’s lack of motivation in doing the exercises from a normal perspective:

He finds excuse for things, he’s very good at that a typical man in that respect. (Carer)

### Relative Experience

Stroke was a relative experience for the participants. Participants expressed different aspects of their experiences of stroke during the narrative interviews comparing their present circumstances with their pre-stroke situations. They used relative mechanisms to compare their situations with the past or with other people whom they knew or with the general public. For example, a stroke survivor describes how life was before the stroke:

I was a very very fit woman I was 52 but I played badminton every week, I used to walk to work every day, I went two or three times a week swimming. I used to walk a lot with the dog. (Stroke survivor)

A 25-year-old student describes her mum’s old times and compares it with her present circumstances:

She used to be a big gardener she got an allotment (community garden). she used to be part of a dance group they did tap dancing they did it for charity, she used to do yoga as well so yeah she was very active, she can’t manage her own life like me and my sister had to take over the financial side of the things such as paying bills which she used to be able to do it herself. She was quite a private person. She was really independent before yeah that has changed. (Carer)

A 59-year-old retired teacher compares her situation with others and perceives herself lucky after the stroke:

I’m lucky I think because I’ve got my daughters to help me yeah, I’m lucky because I enjoy sunshine outside and I’m lucky because of my daughters so I’m alive. (Stroke survivor)

Another 22-year-old stroke survivor describes how he used to go out most nights drinking or in his words “men socializing” and now he has to stop this:

Before I used to go out most nights drinking “men socializing” and going down town just basically keep continuously. I have to stop all that because with tablets and my injections I’m on now. (Stroke survivor)

He replaces his old hobby with a new one stated in the following extract:

I’ve got this just really started and one that I haven’t done before and that’s coin collecting. That’s for old people but never bother and I don’t know why I’ve started doing it. Yeah, instead of sitting and being on the Internet all day, instead of just being on Facebook or playing poker on Internet, I can actually have something to deal with, looking for some coins or if I’ve got a coin then try to research it online. (Stroke survivor)

### Changed Bodies

The focus of participants’ narrative accounts was on the body and how the changed body changed their relationships with others and transformed identities and roles in their social context.

All participants experienced changes in their bodies soon before hospitalization and after being diagnosed with the stroke. The changed body has a direct relationship to the individual’s physical and functional limitations. The changed body also underpins many different aspects of the participants’ experiences such as their relationships with others, their roles and identities, their psychological well-being, their jobs, their coping abilities, and management capabilities. Thus, the experience of transition back to home and to the community is greatly influenced by the participants’ bodily changes post stroke.

One of the participants explains how the changed body has affected her roles and her relationship with her family:

I had the operation and when I had the operation gradually I lost all my left side use of it. I gave up working because I was a teacher I needed to be on my feet so I took an early retirement. My kids, my family are not close anymore at all. It’s just as if they don’t like it (the changed body). I don’t know whether it upsets them see this (referring to the affected hand) I don’t know (long pause). People are better when they get to know you, it’s not understanding it’s empathy. People don’t seem to be able to, they see me as I must’ve done whatever which I didn’t. My body was a very active body. I’d like to be able to get some activities into it. I feel better when I’ve been swimming. I have found the biggest thing is that people now treat me as old. People see me as old. I was never old. Now people see me as OLD. I’m an old woman.

Interviewer: Why? Can you explain please?

I don’t know. I could’ve kept my intelligence which I have and I have done all the tests and people think you’re daft. They talk funny to you and the carers call you darling. Hello darling and how are you today sweet heart. No go away, I will not be patronized. I always say you can insult me if you like but don’t patronize me because I’ve got it. I mean my left arm and left leg don’t work properly but otherwise if you could do *The Times* crossword which I can do I think you’re not lost a lot up here. I won’t have it I won’t be. I am determined I will live normal life. I am utterly determined that’s what I’ll do. I have learned to swim. I can swim. I can’t walk very fast but I can swim. (Stroke survivor)

Another stroke survivor had a different experience of bodily changes. More than two years following the stroke, she does not have major physical disabilities but she perceives her post-stroke body differently compared with her body before stroke. Contrary to most people who experience physical changes in their bodies poststroke, she says,

It’s like it (stroke) hasn’t done to my mobility a lot in comparison with people but I get my words mixed up and I can’t think much and I know I’m not as quick as I used to be and it’s hard. (Stroke survivor)

Despite having no visible body changes, she describes how people perceive her differently following the stroke, the same experience that the first survivor describes in her account:

No good saying it but family don’t understand family don’t understand. They think you sit there and I think this way unless you’re laid in bed poorly I think that’s the only time that people think that there is anything wrong with you, while you are up and about and doing bits and whatever they think you’re ok you know. They think nothing’s changed and you’ve just got to wait on them and things like that. Families don’t and friends don’t because you look ok and you’re walking about and you look all right and people an old fashion says my mum used to say you don’t judge a book by its cover. I don’t have the confidence to go with friends because you don’t know if they’ll understand you if you’re not well. (Stroke survivor)

One of the stroke survivors and his carer had a shared understanding of the changed body and its physical vulnerabilities after stroke. In their jointly produced narrative account, they described how long it took for them to get used to it. The stroke survivor says,

You found ways round it. I used a tray last year when you (his wife) were ill, carry things around the house that was one-handed tray. (Stroke survivor)

They are now so used to the changed body and its functioning that the carer claims,

Now what would happen if his left arm started working again you know because he is so used not to and do everything with one hand and he just adapts. (Carer)

One stroke survivor described a limiting body soon after she woke up in the hospital:

I couldn’t talk, I couldn’t move, nothing.

She experienced the same body as the dependent body after being discharged from hospital and coming home:

I found out I was very ill and very tired, I couldn’t move at first, I couldn’t go upstairs and my daughter had to do everything.

After two years, she still experiences a needy and unreliable body that shapes her relationship with her daughters:

I can’t have drink on my lap because if I might sneeze or something and I can’t talk always but mentally I’m the same, again I’m lucky because my daughter does the cooking. I’m lucky, I think because I’ve got my daughters to help me yeah.

The changed body and the subsequent changed relationships have created negative psychological states such as frustration, stress, and guilt, as her daughter and carer expressed:

She wants to take control of her life, then if it’s a bad day we are back to the beginning if you know what I mean so it’s very frustrating. I must admit. I know my mum feels quite guilty about it so it’s hard to explain. (Carer)

Some participants struggle to overcome the physical limitations of the stroke and it appears that being focused on the uncontrollable aspects of stroke has intensified their negative feelings.

One stroke survivor describes his experience of bodily changes as:

It feels to me that I’ve been playing backwards, there is lot of stuff I can’t do.

He describes a limiting and tiring body that needs help and support to function:

There are lot of things all the time like walking around shopping centers, I can’t. I’ve done once but I was knackered by the end of it. So I rely on wheelchairs now or get pushed around on my scooter.

One of the carer participants interprets the limiting body in terms of the lack of motivation and relates it to laziness. She believes that her partner does not see point in wearing the walking aid technology after the stroke:

He could go for a walk and then start doing something else, he’s kind of all good in theory when he puts it into practice he can’t bother, truthfully. (Carer)

### Change of Perspectives

The experience of stroke creates a situation in which people revise their identities and position themselves in relation to others differently. Over the course of the narrative interviews, they struggled to describe their changed selves, from an active self who was in control in the past to a self who has more passive role in life following the stroke.

One of the stroke survivors in this study links her account on the changed body to the one that describes her relationship with others post stroke. She believes that people’s perception of her is formed by her changed body despite her personality and her mental capabilities. The changed body led to more isolation and portrayed her as old and less intelligent.

It is evident from the account of another survivor that stroke has changed her relationship with friends and family. She struggles to convince others that she cannot do a lot of things as she used to. This is really stressful for her because she cannot accept that she is not able to accomplish her responsibilities as wife and mother, particularly as an independent person in the past who never relied on anybody to do anything.

One of the carer participants expressed her role following her husband’s stroke:

Of course I had to take on a lot more things within the house you know and doing all you know the chores really. Lots of stuff that if he (her husband) had been fit I wouldn’t have done but usually he’s over my shoulder telling me how to do it. I mean I’m perfectly capable of doing it it’s just that’s different it’s now sort of my responsibility mostly. (Carer)

She felt some changes in roles and responsibilities, which have been forced to her because she has been “the fit one.” Generally, she feels all right about this most of the time but she says,

You know like anybody and everybody you get your moments when you think oh I wish I didn’t have to do all of these.

Another carer portrays her relationship with her mum after the stroke and describes how their roles have changed:

I think it’s (the relationship) changed because me and my sister are like parents and she’s like the child I’d say (laughs) because she was really independent before yeah that has changed. I’d say we are like the parents now. She cannot manage her own life like me and my sister had to take over the financial side of the things such as paying the bills which she used to be able to do it herself. She was quite a private person and it’s becoming stressful because it’s very intrusive when an outsider comes and takes over everything but she wasn’t able to deal with that side of things and gradually she’s getting there but still me and my sister have to take over that aspect so that’s been a big burden. (Carer)

## Discussion

The purpose of this article is to use a narrative approach to understand participants’ experiences of living with stroke. Narrative inquiry has been used to explore the complexity of living with stroke including the experiences of embodiment in stroke survivors within the context of their illness (van der Riet, Dedkhard, & Srithong, 2011). The narrative accounts of participants in this study are mainly structured based on how their changed bodies post stroke changed their identities and roles and consequently their relationships with others. The findings showed that they reinterpreted their life as a result of living with stroke and created new meanings for their experiences within their current circumstances (Mishler, 2006; Nasr, Enderby, & Parry, 2012).

Participants in this study created a detailed narrative about their changed bodies. Body and self are considered as a unit, which influence each other in a mutual way. The unit starts to separate when the body is unable to function according to one’s will and desire (Corbin, 2003). That happens for people after a stroke when they attempt to construct their identity in the light of the illness. Body image is the core aspect of identity and “acts as a standard that influences not only the way we think of ourselves, but also our ability to perform various activities and the goals we set for the future” (Chrisler & Ghiz, 1993, p. 68). For some stroke survivors, the convergence of body and self is a great challenge, as it is expected in long-term conditions. They constantly see a differentiation of body and self, which has affected their roles and their relationships with families and friends. According to Fox and Ward (2006), “body/self comes into being only in its relations to physical forces of nature, to cultural and social expectations and constraints, emotional attachments and reflexive understandings” (p. 475). For example, the limiting body of one of the participants is not able to represent her as a teacher and therefore forced her to retirement. Her changed body could not cope with an active lifestyle as was expected by her partner and its appearance created problems in her relationships with her children. Hence, a struggle is seen to free the self trapped in the changed body and to construct meaning of life around what the self expects. The loss of skills and activities that stroke survivors used to do before the stroke greatly influences their sense of identity that remains with them years after the stroke and creates a distance between them and their new selves (Murray & Harrison, 2004).

After a few years post stroke, they might get familiar with their strange bodies but how the body is perceived by others reinforces the separation of the body and self and acts as an obstacle to the emergent body/self.

In the narrative account of one of the stroke survivors, body and self take on another dimension. The stroke survivor has experienced severe bodily changes. He is now so used to the changed body and has become so familiar with it. It seems that he recognizes the changed body over time. In other words, he “re-owns” his changing body and feels at ease with it (Kvigne & Kirkevold, 2003). He integrates the paralyzed arm and leg and makes meaning of the self. He uses a range of tools and mobility aids from electric scooter to one-handed tray and constructs meaning of the use of aids by normalizing them and putting them in the broader mainstream picture.

Focusing on bodily changes has also been documented in other studies. Kirkevold (2002) showed that stroke patients experience bodily changes at the onset of their journey, during the acute phase and hospitalization, and at home where they experience bodily changes in a more natural environment while trying out day-to-day activities of life. Eilertsen, Kirkevold, and Bjork (2010), in a study of Norwegian women, showed the experience of the bodily changes over the first two years post stroke. Participants experienced unpleasant bodies when they were in hospital and “adopted a wait-and-see approach” with an expectation that the body would improve. When returned home, they were challenged with the practicalities of performing daily tasks in a less controlled situation where they experienced unpredicted and unreliable bodies. In general, the experience of unpleasant, unpredictable, unreliable, betrayal, and dysfunctional body created a sense of bodily strangeness. During the second year of stroke, however, they shifted their focus from their bodies and their functional limitations to other meaningful aspects of their life with the realization that the bodily changes could be permanent.

In the present study, the construction of identity whether it was the outcome of the confluence of body and self or it was the effect of the body/self division was grounded in participants’ experiential knowledge. They did not provide medical narratives concerning the diagnosis, medical treatment, and rehabilitation of the stroke, and their identities were not located in scientific explanatory knowledge.

As pointed out earlier, chronic illness models are predominantly used to explain the experiences of living with long-term conditions. The redefinition of life during a long-term condition is also reflected in chronic illness models. Corbin and Strauss’s (1992) chronic illness trajectory framework consists of several phases reflecting the endless and continuous process of revising the experience, coping, and adaptation to the stressful situation. The model aims to map between the patient’s life story/biography and interventions, which requires deep understanding and detailed knowledge of the individual’s biography as well as the impact of social and environmental factors and their possible negative and restricting effects on the disease process (Burton, 2000).

Becker (1993), in a study of stroke patients, explained the notion of continuity as a symbolic process through which individuals create fluid identities for themselves and construct a sense of continuity by reinterpreting and revising life events. Although the symbols of continuity might remain unchanged, their manifestation might change. Becker concludes that understanding how people redefine life experiences following a disruption such as stroke and how they use the stressful and disrupting stroke to recreate continuity would open the door to facilitate care during the process of rehabilitation and could inform policy and practice in the management of long-term conditions (Becker, 1993).

The concept of “biographical disruption” (Bury, 1982), however, was challenged by Faircloth, Boylstein, Rittman, Young, and Gubrium (2004) in a qualitative study of stroke survivors in which in-depth interviews were conducted with the participants at five intervals over time. The findings which are in line with the findings of the present study showed that stroke survivors perceived the stroke as a normal component of aging and linked it with their preexisting co-morbidity. In addition, their prior knowledge of stroke as being a stroke survivor or having cared for a person with stroke lessened the disruptive effect of stroke in their life. Having related their findings to the concepts of “biographical reinforcement” and “biographical continuity” (Carricaburu & Pierret, 1995), Faircloth and colleagues (2004) concluded that the “biographical disruption” model cannot adequately explain the experiences of stroke, and they suggested that rather than disrupting a biography, an illness such as stroke can construct a biography that flows across time and space and therefore they supported the idea of “biographical flow” (p. 256).

## Implications and Limitations

The adopted methodology and the findings of this study provided us with an understanding of the experiences of stroke survivors in their social and personal contexts. The findings offered windows through which we could watch for the impact of contextual factors when developing interventions for long-term conditions. For example, the findings could enlighten the process of design and development of self-management interventions for stroke where the significance of personal experiences of stroke should be reflected in these interventions and their components should be aligned with the individual’s experience of stroke and its impact on their life and their stroke journey. One of the limitations of the study was that the sample was small, which would make the findings prone to bias if data had been aggregated for statistical analysis or data saturation. However, the participants provided us with their unique experience of living with stroke through the method of narrative and storytelling. Their individual stories were never treated as representative data or were never been used to reach data saturation.

## Conclusion

We used a narrative approach to understand the experiences of living with stroke. Stroke was perceived by the participants as a sudden relative event that should be normalized to fit into their life and its meaning is appraised by comparing participants’ situation with the past or with other people. The findings also showed that following stroke people experience bodily changes and these changes have great impact on their relationships and personal and social roles. The methodology and findings of this study are a timely reminder of how the growing body of qualitative research of illness can add valuable perspectives to research projects developing interventions for the management of long-term conditions.
